# Quality of care in sickle cell disease: Cross-sectional study and development of a measure for adults reporting on ambulatory and emergency department care

**DOI:** 10.1097/MD.0000000000004528

**Published:** 2016-09-02

**Authors:** Christian T. Evensen, Marsha J. Treadwell, San Keller, Roger Levine, Kathryn L. Hassell, Ellen M. Werner, Wally R. Smith

**Affiliations:** a American Institutes for Research, Chapel Hill, NC; b Department of Hematology/Oncology, University of California San Francisco Benioff Children's Hospital Oakland, Oakland, CA; c Redwood City, CA; d Division of Hematology, University of Colorado, Aurora, CO; e Blood Epidemiology and Clinical Therapeutics Branch, Division of Blood Diseases and Resources, National Heart, Lung, and Blood Institute, Bethesda, MD; f Division of General Internal Medicine, Virginia Commonwealth University, Richmond, VA.

**Keywords:** access to care, ambulatory care, emergency medicine, health care delivery, quality of care, sickle cell disease

## Abstract

Documented deficiencies in adult sickle cell disease (SCD) care include poor access to knowledgeable providers and inadequate treatment in emergency departments (EDs).

The aim of this study was to create patient-reported outcome measures of the quality of ambulatory and ED care for adults with SCD.

We developed and pilot tested SCD quality of care questions consistent with Consumer Assessments of Healthcare Providers and Systems surveys. We applied psychometric methods to develop scores and evaluate reliability and validity.

The participants of this study were adults with SCD (n = 556)—63% aged 18 to 34 years; 64% female; 64% SCD-SS—at 7 US sites.

The measure used was Adult Sickle Cell Quality of Life Measurement information system Quality of Care survey.

Most participants (90%) reported at least 1 severe pain episode (pain intensity 7.8 ± 2.3, 0–10 scale) in the past year. Most (81%) chose to manage pain at home rather than the ED, citing negative ED experiences (83%). Using factor analysis, we identified Access, Provider Interaction, and ED Care composites with reliable scores (Cronbach α 0.70–0.83) and construct validity (*r* = 0.32–0.83 correlations with global care ratings). Compared to general adult Consumer Assessments of Healthcare Providers and Systems scores, adults with SCD had worse care, adjusted for age, education, and general health.

Results were consistent with other research reflecting deficiencies in ED care for adults with SCD. The Adult Sickle Cell Quality of Life Measurement Quality of Care measure is a useful self-report measure for documenting and tracking disparities in quality of SCD care.

## 1 Introduction

The probability of survival to adulthood in sickle cell disease (SCD) has improved to nearly 95% in the United States^[1]^ with a range of therapies preventing early mortality.^[2–5]^ As life spans increase, adults with SCD face increased morbidities including multiorgan failure, chronic pain, and neurocognitive deficits.^[6–8]^ The growing demand for health care for adults with SCD reveals inadequacies, including limited access to providers who are knowledgeable about unique clinical needs of the population.^[6,9]^

Adults with SCD report stigmatization in the health care system^[10–13]^ with providers generally insensitive to the pain experiences characteristic of SCD. Providers can be overly concerned about addiction, leading to failure to provide timely and adequate pain control when needed.^[12,14–16]^ In turn, these negative health care experiences were related to many adults postponing seeking health care, managing pain episodes at home, and self-discharging from the hospital.^[17–20]^

As a consequence of these shortcomings, the National Heart, Lung, and Blood Institute (NHLBI) launched a series of efforts aimed at improving adult SCD care, including the recent development of a set of evidence-based clinical practice guidelines for primary care providers.^[21]^ NHLBI also sponsored the development of the Adult Sickle Cell Quality of life Measurement (ASCQ-Me—pronounced “Ask me”) information system,^[22,23]^ a system designed to be complementary with the Patient-Reported Outcomes Measurement Information System (PROMIS).^[24]^ ASCQ-Me and PROMIS (a National Institutes of Health Common Fund initiative) use comparable methods for instrument development and validation to create state-of-the-science assessments for self-reported health.^[25]^

As part of the ASCQ-Me project, we concurrently developed and tested the ASCQ-Me Quality of Care (QOC) survey to be a patient-reported outcome designed to assess access and quality of adult sickle cell care. We used rigorous psychometric methods to evaluate the ASCQ-Me QOC survey with a cohort of adults with SCD, using a cross-sectional design. Our goal was to develop a measure that allows for comparison of results across care settings and over time, and to produce comprehensible information that has utility for both health care providers and patients served.

Here we describe the development and psychometric properties of the ASCQ-Me QOC survey. We hypothesized that responses of adults with SCD to a series of questions about their health care experiences could be used to derive reliable and valid QOC scores. Moreover, we expected that the empirical evidence provided by these questions would reliably discriminate the care experience of patients with SCD from that of other populations.

## 2 Materials and methods

All materials, methods, and procedures received approval from the Institutional Review Boards of the American Institutes for Research, Children's Hospital Oakland Research Institute, and the sponsoring hospitals.

### 2.1 Participants

Participants completed the ASCQ-Me QOC survey anonymously during the ASCQ-Me field test data collection, a larger study with methodology detailed elsewhere.^[22,23]^ The sample size requirement for the QOC analysis was based on the number of respondents per question. Depending on the intercorrelations among items in the factors, recommendations can vary from 5 to 20 respondents per item in the factor analysis to support stable estimates—larger sample sizes are required when the intercorrelations are smaller.^[26]^ There were 12 questions hypothesized to address 4 composites in the field test version of the ASCQ-Me QOC; thus, a sample size of 240 would be ample. The targeted enrollment for the ASCQ-Me field test of 550 adults far exceeded that needed for the current study.

Data were collected at 7 geographically and clinically (e.g., academic medical center, community-based organization, rural health center) varied sites of care, with the goal of addressing potential sources of bias by enrolling a diverse sample of patients. Inclusion criteria were broad, as follows: any diagnosis of SCD and ages ≥18 years. Exclusion criteria were as follows: no diagnosis of SCD or diagnosis with sickle cell trait, younger than 18 years, and not able to read English. Participants answered screener questions when responding to recruitment materials in order to determine that they should not be excluded. Their sickle cell providers also referred participants to the study so they had a known diagnosis of SCD. Data were collected between October 2008 and May 2009.

### 2.2 Data collection procedures

Participants were volunteers responding to descriptions of the study at clinical care sites, SCD community-based organizations, and the Sickle Cell Disease Association of America website. At ASCQ-Me field test sites, site coordinators explained study procedures in detail to qualified adults who then signed informed consent forms. Site coordinators then entered minimal demographic data about the participants (age range, gender, and diagnosis) and assisted them in logging into the ASCQ-Me data collection website. Participants proceeded to complete the survey on their own.

### 2.3 Measurement development

We generated preliminary themes about the experiences of adults with SCD, and then ASCQ-Me questions, from information from the NHLBI consumer working groups^[9]^ and a literature review.^[22]^ Next, we conducted individual and focus group interviews involving 122 adults with SCD and 15 providers. We qualitatively analyzed participant responses to confirm and expand on initial themes related to QOC.^[22]^ Themes identified included the following:

1. Lack of SCD knowledge on the part of providers in ambulatory and emergency department (ED) settings, leading to stigmatization of adults with SCD as drug seeking, as well as to inappropriate and ineffective care
2. Extremely long waits before receiving care in the ED
3. Disrespectful providers who did not consider information provided by the patient, leading to undermedication or overmedication for pain
4. Preference of adults to treat the symptoms of SCD outside of the health care setting, given the preceding

These themes informed the construction of the ASCQ-Me QOC survey questions that were also modeled after the Consumer Assessments of Healthcare Providers and Systems (CAHPS) surveys.^[27,28]^ CAHPS represents the most widely used method for capturing and reporting patients’ experiences of their care, covering literally millions of lives. CAHPS measures are used to assess the performance of health care entities including Medicare, Medicaid, private health plans, clinicians, and medical groups, among others.^[29–33]^ We included questions that were similar or identical to CAHPS when questions were consistent with content suggested by the ASCQ-Me formative research, in order to enable comparisons across care experiences.

The ASCQ-Me QOC survey consists of 27 questions, but skip patterns allow respondents to complete the survey in as few as 5 questions, if they did not have any sickle cell–related pain in the past 12 months and never sought emergency or ambulatory care. Twelve ASCQ-Me QOC questions were hypothesized to be indicators of 4 domains of health care quality. The first domain mirrors the CAHPS “Access” composite^[34]^ and consisted of 2 items, for example, “In the past 12 months, when you tried to make an appointment to see a provider, how often were you able to get one as soon as you wanted?” (rated *Never* to *Always*). The second domain mirrors the CAHPS “Provider Communication” composite^[34]^ and consisted of 4 items, for example, “In the past 12 months, how often did the doctor or nurse listen carefully to you?” (rated *Never* to *Always*). The third domain, “ED Care,” included 3 items regarding the patient's interaction with staff (doctors, nurses, clerks/receptionists) during emergency visits along with an item asking the extent to which providers believed that the patient had severe pain.^[34]^ The fourth composite—ED Pain Treatment—included 2 items that asked how successfully pain was treated in the ED, and how long the patient had to wait to get treated.^[34]^

Three questions addressed global evaluations of care for SCD by asking participants how often they were satisfied (*Never* to *Always*) with their usual provider, and with the QOC received from their usual provider and the ED. Participants also provided an overall evaluation of all the care they received on a scale from 0 to 10 anchored from *Worst* to *Best care possible*. Separate scores were produced for each of the 4 global questions.

For the preceding 12 months, participants reported on the number of visits with their usual provider and the number of times they had been to the ED because of pain or had managed pain at home, without going to a doctor, clinic, or hospital. Three other questions measured participants’ perceptions of how knowledgeable their primary care provider was about SCD and the final 2 questions asked if participants had ever avoided going to the ED when they thought they needed care, either due to bad experiences or due to health insurance issues.

### 2.4 Data analysis

Data analyses were conducted using Statistical Analysis Software (www.sas.com). Twelve QOC survey questions were hypothesized to form 4 composite scores: Access, Provider Communication, ED Care, and ED Pain Treatment. We evaluated the amount of systematic information provided by these 4 composites by determining their internal consistency reliability using Cronbach α.^[35]^ We examined the distribution of composite scores to see the percentage of patients who reported the highest (ceiling effect) and lowest (floor effect) quality. We evaluated the construct validity of the composites using the following: scaling success rate^[36,37]^—to determine whether 100% of correlations between responses to questions and their composite score were stronger (correcting for overlap) than the correlation of those questions with other composite scores^[38]^; confirmatory factor analysis (CFA)^[39,40]^—to determine whether the hypothesized relationships between indicators and underlying dimensions of QOC fit the data by requiring fit statistics >0.95 and the root mean square error of approximation (an indication of the amount of variance in the data not accounted for by specified relationships between questions and composites) <0.06^[41]^; and examination of correlations of each composite measure score to the 4 overall ratings of care.^[42]^

We imputed missing data in order to conduct the factor analysis. We used a multiple imputation procedure. One of the 5 data sets of imputed values produced by missing values procedure was selected at random to be used as input for these exploratory models and the goodness-of-fit analyses (CFA). Once a final revised model was identified, the analyses were repeated on each of the 4 remaining sets of imputed values to evaluate the generalizability of the results. The analyses were then repeated using all 5 sets of imputed values combined into 1 data set. Results from these subsequent analyses are reported only if they differ from the results based on the single set of imputed values and if they suggest a poor fit for the revised structure.

We also examined the validity of ASCQ-Me QOC scores by comparing the scores of those of “known groups”—comparable, publicly reported scores for people covered under private insurance, Medicare, and Medicaid.^[43]^ We expected QOC scores for adults living with SCD to be more similar to those for Medicaid, with lower quality scores compared with Medicare or private insurance.^[22]^

When the CFA did not support the hypothesized relationship of questions to composite measures, we conducted exploratory factor analysis (EFA) and examined the rotated factor loading pattern. We identified those items as belonging to a composite that had a factor loading >0.30, with differences between that primary loading and any secondary loadings on competing factors of ≥0.20.

## 3 Results

### 3.1 Demographic characteristics

A total of 561 adults with SCD at geographically dispersed sites were enrolled in ASCQ-Me with 556 completing the QOC survey. Participants were mostly female (64%) and most were between the ages of 18 and 34 years (63%), with 20% aged 35 to 44 years, and 18% aged 45 years and older. Most participants reported that they had the Hb-SS variation of SCD (64%), followed by Hb-SC (21%), and Hb-Sβ thalassemia (10%). Less than 1% of respondents (0.89%) had missing data on the QOC survey.

### 3.2 Characteristics of pain

Almost all participants (90%) indicated that they had had a severe pain episode in the past 12 months, with 20% reporting a pain episode within the past week, and 9% reporting a pain episode at the time of the survey. Half reported ≥4 episodes and 9% reported no pain episodes in the past 12 months. The average severity rating for the last pain episode was 7.8 ± 2.3 on a word graphic rating scale, where 0 = no pain and 10 = worst pain imaginable. Most participants (67%) reported that the pain was severe enough to interfere with their lives, with 37% reporting that the pain was so severe that they could not take care of themselves and needed either some help or constant care. The duration of the most recent pain episode ranged from 1 day (68%) to >1 week (21%).

### 3.3 Characteristics of participants’ health care

Seven questions on the ASCQ-Me QOC survey were not hypothesized to contribute to composite scores but were included to provide information to characterize health care experiences (Table 1). The majority of participants (89%) reported having a regular provider specializing in SCD care that they saw for routine care, and most (61%) had seen this provider ≥4 times in the past year. Most participants (81%) also reported going to the ED for pain in the past 12 months, with 32% visiting the ED for pain ≥4 times. Waits of >1 hour before treatment were common (reported by 62%). Participants reported treating the majority of pain episodes at home without medical assistance, with 83% reporting that previous bad experiences with ED care played at least some role in that decision.

**Table 1:**
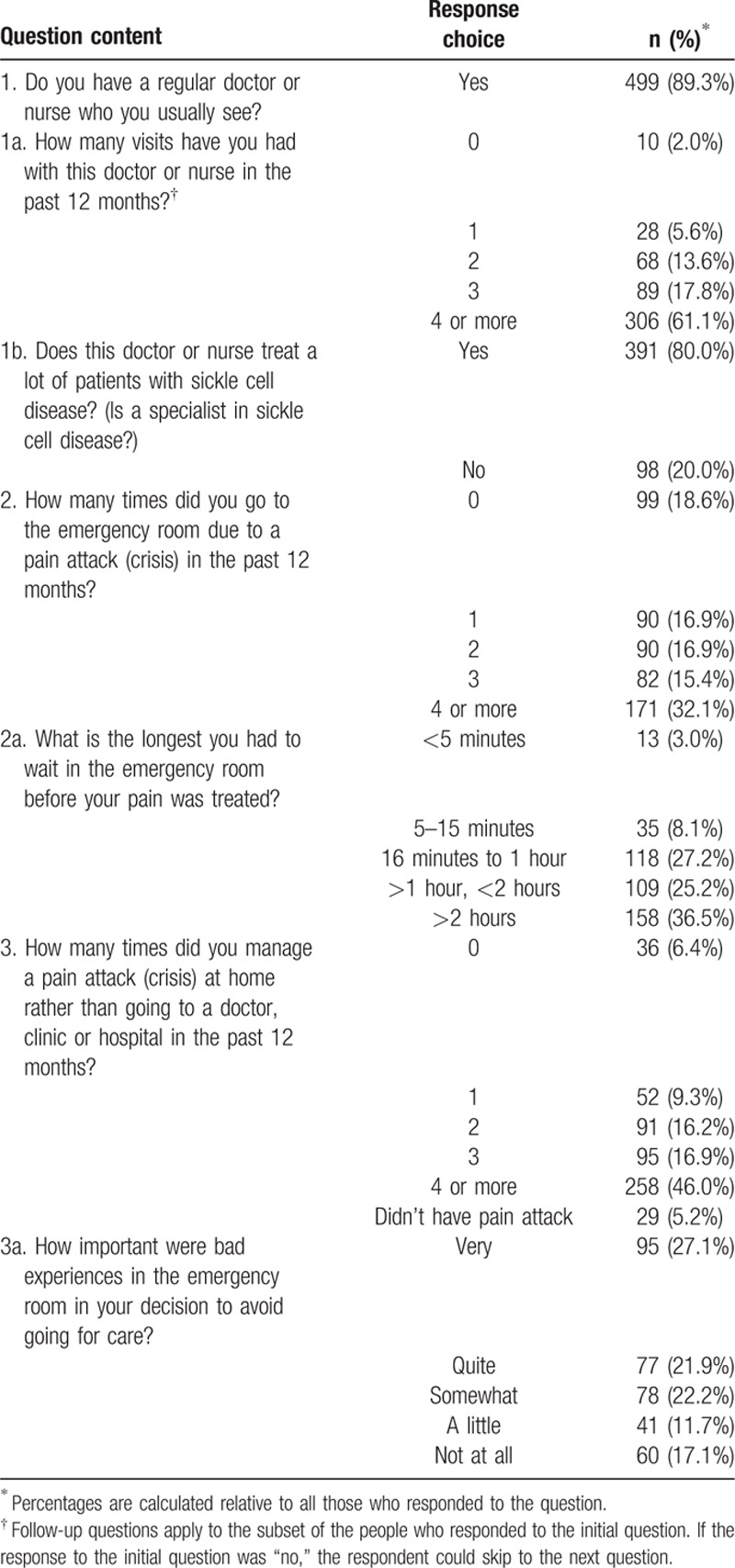
Type of care received: report by adults with sickle cell disease (N = 561).

### 3.4 Reliability and validity of composites

The CFA fit statistics did not support the arrangement of the 12 questions into 4 composites, so we conducted EFA on the questions plus the question on bad experiences in the ED. The EFA pointed toward 3 composites that had fit indices exceeding the conservative cutoff criterion of 0.50 for loading on a single factor and no loadings on a secondary factor >0.25, with the exception of the question about ease of getting an appointment for ambulatory care (Table 2). We labeled the composites Provider Communication (quality of patient and provider communication), ED Care (QOC in the ED), and Access (access to routine and emergency care). We retained the question about access to routine ambulatory care for further analysis because it referred to content that would be important to include in the final survey. Subsequent statistical analysis supported this decision. Once we accounted for residual correlations among items due to inclusion of the phrase “emergency care” in the question stem, the CFA with the 3-factor, 13-question composite structure fit the data very well (CFI = 0.97; root mean square error of approximation = 0.05). Internal consistency reliabilities for all 3 composites were >0.70, and all composite questions were more highly correlated with their own composites versus competing composites (scaling success rate for all 3 was 100%; Table 3). Floor effects were minimal for all composites and ceiling effects were minimal for the Access and ED Care composites (Table 3). More than 40% of participants reported the highest QOC possible for Provider Communication.

**Table 2:**
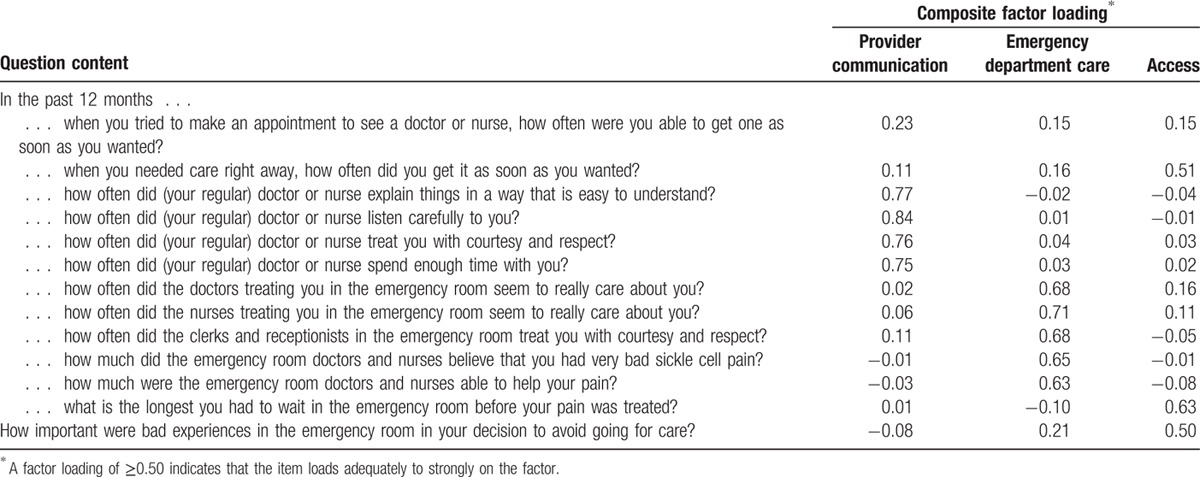
Factor loadings for 13 Adult Sickle Cell Quality of Life Measurement Information System—quality of care questions on three composites.

**Table 3:**
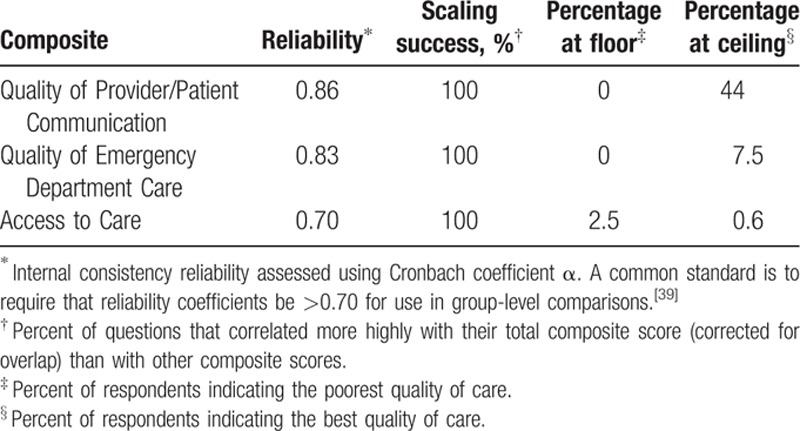
Reliability, scaling success, and distributional properties of composite scores.

Correlations between the composites and the CAHPS global ratings of QOC can be seen in Table 4. Sample sizes associated with different questions differed because respondents were instructed not to answer questions that did not pertain to them. For example, if they did not go to the ED, they were asked to skip the questions asking about their experience in the ED. Stronger relations of the Provider Communication composite with overall ratings of routine care (*r* = 0.65) and provider ratings overall (*r* = 0.83) compared with the other composites provided evidence of construct validity. Similarly, the ED Care composite was most strongly related to overall ratings of QOC in the ED. Interestingly, the Access composite was also most highly related to overall evaluations of ED care.

**Table 4:**
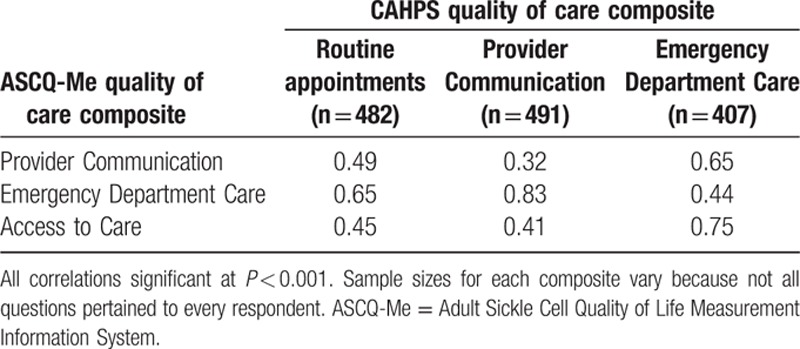
Consumer Assessment of Healthcare Providers and Systems (CAHPS) correlations with quality of care ratings.

**Figure 1:**
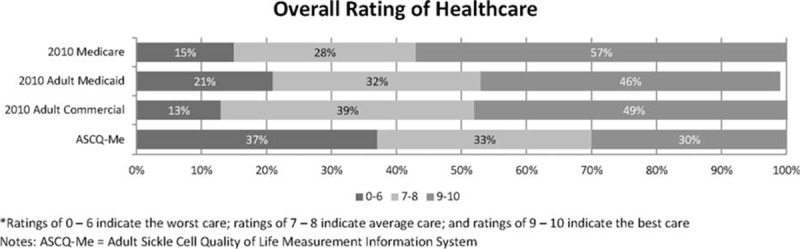
Quality of care of adults with sickle cell disease compared with other populations: overall ratings. ASCQ-Me = Adult Sickle Cell Quality of Life Measurement Information System. Ratings of 0 to 6 indicate the worst care; ratings of 7 to 8 indicate average care; and ratings of 9 to 10 indicate the best care.

**Figure 2:**
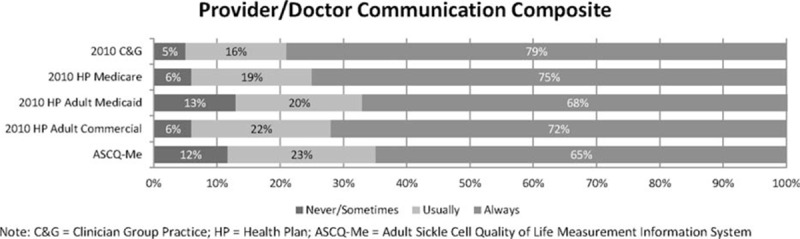
Quality of care of patients with sickle cell disease compared to other populations: Provider/Doctor Communication rating. ASCQ-Me = Adult Sickle Cell Quality of Life Measurement Information System, C&G = clinician group practice, HP = health plan.

Figures 1 and 2 display the frequency distributions of overall ratings of care and quality of communication between patient and provider, respectively, for adults completing ASCQ-Me compared to those of other patient populations who responded to the same questions included in CAHPS surveys. Scores for all groups were adjusted for patient mix using general linear models in which age, education, and general health status were specified as covariates.^[44]^Figure 1 shows that the overall rating of health care by adults with SCD was substantially worse than all other groups, including Medicaid.

Reports of poor provider communication for adults with SCD (Doctor/Provider Communication composite) were comparable to reports of adults treated under Medicaid^[45]^ (Fig. 2). Both groups reported more often that providers “never” or only “sometimes” performed quality communication behaviors, that is, listened carefully, spent enough time, treated them with respect, and explained things well, compared with participants seen in group practices, those in the hospital, those on Medicare, and those on commercial insurance plans.

## 4 Discussion

We propose our successfully developed measure, ASCQ-Me QOC, as a patient-reported outcome measure of QOC for adults with SCD. While the ASCQ-Me QOC survey was developed with 4 domains in mind—Access, Provider Communication, ED Care, and ED Pain Treatment—factor analytic results provided more support for a 3-composite structure in which subsets of questions hypothesized to belong to the ED Pain Treatment topic were found to be highly related to either Access or ED Care. Given the salient role that pain episodes play in most patients’ lives and in their interactions with the health care system, it makes sense that questions referring to SCD pain treatment would not be separable from other aspects of the patients’ interactions with the health care system. Our literature review and qualitative research indicates that the experience of SCD pain pervades all areas of the lives of adults with SCD.^[22]^ Also consistent with this literature, the composite that most strongly related to overall evaluations of health care was ED Care.

Results of our psychometric analyses supported the reliability and validity of the 3 composites—access to care, quality of provider communication, and QOC in the ED—for adults with SCD. All 3 composites showed good precision for discriminating among groups experiencing poor QOC, while the low ceiling effects for the Access and ED Care composites suggest that these measures will do a good job of discriminating among groups of individuals who experience higher QOC. The Provider Communication composite may not be as discriminating among groups of individuals who have positive experiences on this care dimension; however, ceiling effects of ≥44% are not unusual for items that ask patients to evaluate their providers.^[46,47]^ Moreover, Fig. 2 shows that this composite was able to distinguish differences in care provided to adults with SCD from that provided to other groups.

Our hypothesis that adults with SCD would report care experiences similar to those reported by patients treated under Medicaid (who have historically given the lowest ratings of care when compared to patients treated under Medicare or private insurance^[43]^) was supported. These results not only support the validity of the ASCQ-Me QOC survey but are also consistent with studies showing that adults with SCD report greater race-based and disease-based discrimination in health care compared with other African Americans.^[48]^ In turn, negative provider attitudes influence patient–provider interactions and quality of SCD care provided.^[49]^

The majority of our volunteer sample of adults with SCD had reasonable access to ambulatory care, which is not surprising given our sampling strategy that was appropriate for conducting psychometric evaluation of the ASCQ-Me QOC survey and that was not intended to be representative of the population of adults with SCD. Our select group of adults with SCD reported that their outpatient providers were knowledgeable about SCD care and highly rated regarding communication, respect, and time spent. Nevertheless, our adults reported very poor QOC in the ED. The majority of adults with SCD in the United States do not have access to either adequate ambulatory or adequate ED care.^[15,20,22]^

Because the ASCQ-Me QOC survey psychometrics were generated within the context of a larger field test including ASCQ-Me short forms and PROMIS measures, we did not time how long it took respondents to complete the QOC measure. Given its length, the time to complete the ASCQ-Me QOC survey is likely to be <10 minutes, according to previous research and practice, with quicker times expected if the survey is administered electronically.^[50,51]^ Given skip patterns, the fewest number of questions that an adult with SCD would have to answer would be 5, thus taking <2 minutes to complete. This brevity lends to the use of the ASCQ-Me QOC survey in day-to-day clinical practice.

The needed improvement of adult health care for SCD is literally a matter of life and death. In our qualitative data analysis for ASCQ-Me, adults reported that they delayed or completely avoided going to the ED because of their past negative experiences, despite facing life-threatening complications.^[22]^ Deaths related to SCD due to patient refusal to seek needed health care have been documented.^[52]^ Severe painful vaso-occlusive episodes remain a marker for premature mortality in a modern cohort^[53]^ and the well-documented delays in treatment when adults with SCD present to the ED^[54]^ can be associated with preventable deaths, such as what can occur with the rapid progression of acute chest syndrome.^[8,55]^ The research reported here suggests that the ASCQ-Me QOC survey could assist in efforts to improve the QOC delivered to adults with SCD by identifying deficiencies from the patient's perspective and enabling care delivery systems to implement quality improvement initiatives and evaluate their success in addressing these deficiencies.

## 5 Conclusion

Understanding patient experiences is a first step in improving the QOC delivery. Improving access to routine and emergency care, provider communication, and the quality of emergency care as measured by the patient-reported outcome measure, ASCQ-Me QOC, can potentially reduce health care costs as patients receive timely and appropriate care.^[56]^
